# Appressorium: The Breakthrough in *Dikarya*

**DOI:** 10.3390/jof5030072

**Published:** 2019-08-03

**Authors:** Alexander Demoor, Philippe Silar, Sylvain Brun

**Affiliations:** Laboratoire Interdisciplinaire des Energies de Demain, LIED-UMR 8236, Université de Paris, 5 rue Marie-Andree Lagroua, 75205 Paris, France

**Keywords:** appressorium, infection cushion, penetration, biomass degradation, saprotrophic fungi, *Eumycetes*, cellophane

## Abstract

Phytopathogenic and mycorrhizal fungi often penetrate living hosts by using appressoria and related structures. The differentiation of similar structures in saprotrophic fungi to penetrate dead plant biomass has seldom been investigated and has been reported only in the model fungus *Podospora anserina*. Here, we report on the ability of many saprotrophs from a large range of taxa to produce appressoria on cellophane. Most *Ascomycota* and *Basidiomycota* were able to form appressoria. In contrast, none of the three investigated *Mucoromycotina* was able to differentiate such structures. The ability of filamentous fungi to differentiate appressoria no longer belongs solely to pathogenic or mutualistic fungi, and this raises the question of the evolutionary origin of the appressorium in *Eumycetes*.

## 1. Introduction

Accessing and degrading biomass are crucial processes for heterotrophic organisms such as fungi. Nowadays, fungi are famous biodegraders that are able to produce an exhaustive set of biomass-degrading enzymes, the Carbohydrate Active enzymes (CAZymes) allowing the potent degradation of complex sugars such as cellulose, hemicellulose, and the more recalcitrant lignin polymer [1]. Because of their importance for industry and biofuel production in particular, many scientific programs worldwide aim at mining this collection of enzymes in fungal genomes and at understanding fungal lignocellulosic plant biomass degradation. *Podsopora anserina*, a saprotrophic coprophilous *Ascomycota* from the *Sordariales* order, emerged as a perfectly well-suited genetic model system to study plant biomass degradation. This potent cellulose degrader is also likely able to degrade lignin [2,3,4].

In contrast to saprotrophic fungi, most mutualistic or pathogenic fungi must penetrate their host to access nutrients, which explains why these fungi have developed sophisticated tools allowing the attachment, lysis and mechanical breaching of the host surface—three mandatory steps determining host invasion. The best characterized penetration structure is the “unicellular” appressorium, first described in 1883 by Frank in the plant pathogen *Colletotrichum lindemuthanium* as the “adhesion organ” [5,6]. For the sake of simplicity, and in keeping with Emmett and Parbery’s definition of the appressorium which was synonymous with “the infection structure” regardless of its morphology [7], we will also use appressorium to refer to the “appressorium-like“ structures described in species such as *Botrytis cinerea* or *Podospora anserina* [8,9]. The appressorium has been thoroughly studied in plant pathogen species such as *Magnaporthe oryzae* or *Ustilago maydis* [10] and it has also been described in entomopathogenic fungi like *Beauveria bassiana*, in mutualistic arbuscular mycorrhizal fungi of the *Glomeromycota* phylum, and in phylogenetically distant *Oomycetes* including *Phytophthora infestans* [11,12,13]. It is noteworthy that species like *Rhizoctonia sp*., *Sclerotium sp*. (*Basidiomycota*), *Sclerotinia sp*. and *B*. *cinerea* (*Ascomycota*) can develop multicellular penetration structures called infection cushions to penetrate their host [7,14].

Whether saprobes develop penetration structures or not has long been ignored, probably because, at first glance, their saprotrophic lifestyle does not require any host penetration step in order to access nutrients. Recently, we have discovered that the saprotrophic fungus *P*. *anserina* is able to differentiate appressoria when grown on cellophane [8]. Moreover, we have shown that the genetic program controlling appressorium development in this model fungus shares common components with the one of the phytopathogenic fungi *M*. *oryzae* and *B*. *cinerea*. In these three species, the superoxide (O_2_^−^)-producing enzymes Nicotinamide-Adenine-Dinucleotide-Phosphate (NADPH) oxidases Nox1 and Nox2, together with their regulatory subunits NoxR, NoxD, the Pls1 tetraspanin as well as the Mitogen-Activated-Protein-Kinase (MAPK) pathway Fus3/Mpk2/Pmk1, are major components of appressorium development [15,16,17]. This leads to the conclusion that appressoria in saprotrophic and phytopathogenic fungi may be homologous structures. This hypothesis prompted us to investigate appressorium development in various saprotrophic *Eumycetes*. For this purpose, we assessed appressorium development in a fungal collection containing 38 strains grown on cellophane. The studied filamentous fungi included *Pezizomycotina*, *Agaricomycotina* and *Mucoromycotina*. In *Pezizomycotina* especially, this collection allowed us to test every main subphylum except the *Lecanoromycetes*, which are exclusively found in lichens and the *Laboulbeniomycetes*, which are obligate insects parasites, neither of them being easy to culture in the lab. When available in our collection, we investigated different isolates from the same species and different species from the same genus, in order to determine the versatility of the appressorium development capacity at the species level and at the genus level respectively. Finally, since *Sordariomycetes* include a majority of species of particular interest (i.e., *Trichoderma* spp., *Podospora* spp., *Neurospora crassa* and *Sordaria macrospora*), we investigated this class more thoroughly.

## 2. Materials and Methods

### 2.1. Strains and Culture Conditions

The strains used in this study are all listed in Table S1. Each species was cultured on standard M2 medium topped with a single cellophane layer (Bio-Rad n°cat 1650963, Hercules, CA, USA) at 27 °C. The composition of the M2 medium is available on the website (http://podospora.i2bc.paris-saclay.fr/methods.php#cultivate). Depending on the mycelial growth rate of each fungal strain, microscopic observations were carried out between 3 and 15 days after inoculation.

### 2.2. Spores Harvesting and Inoculation

Fungi were grown on M2-standard-medium petri plates till confluence. The plates were flooded with 0.05% tween-water to obtain a spore suspension. Spores were filtered through Miracloth (Merck Millipore, Burlington, MA, USA) and counted. A volume of 100 µL of a 10^4^–10^5^ spore.mL^−1^ suspension was deposited on a cellophane layer (on top of M2 medium). Post-germination mycelial growth was checked for appressorium development from 16 to 30 h post-inoculation.

### 2.3. Microscopic Observations

**Figure 1:**
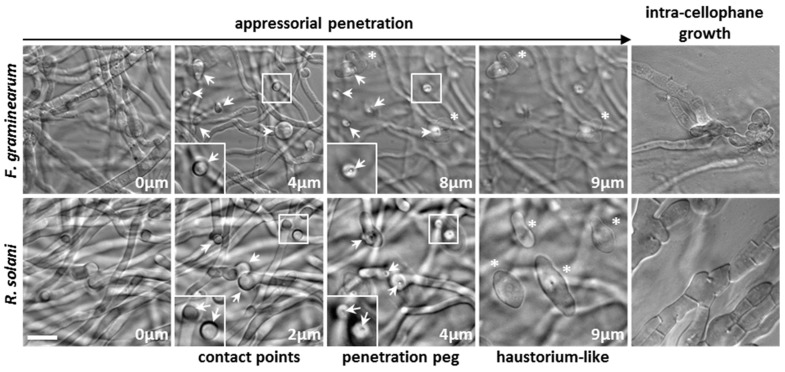
*Fusarium graminearum* and *Rhizoctonia solani* penetrate cellophane through appressoria. For both *F*. *graminearum* (**upper** panel) and *R*. *solani* (**bottom** panel), the main steps of appressorium-based cellophane penetration have been represented. From left to right, the four first columns represent successive z-planes of the same field of view. The distances from the initial z-plane are indicated. Arrows indicate contact points and the penetration peg they emit to penetrate cellophane. (*) Haustorium-like growth. Last column (**right**) shows in-depth intra-cellophane mycelial development. Scale bar = 10 µm. Magnification in the bottom insets is 1.7×.

**Figure 2:**
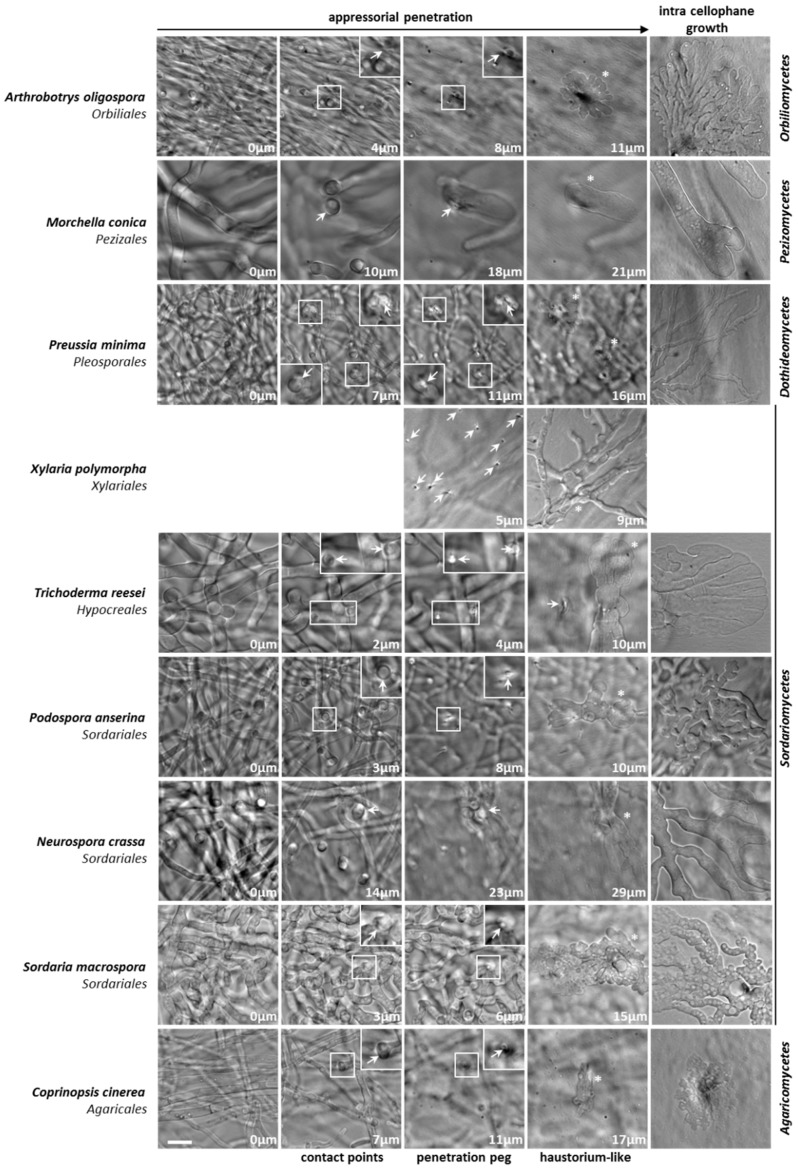
Appressoria in *Dikarya*. For a selection of the representative species of the main groups of fungi endowed with an appressorium to penetrate cellophane, the main steps of appressorium-based cellophane penetration have been represented. From left to right, the first four columns represent successive z-planes of the same field of view. The distances from the initial z-plane are indicated. For *Xylaria polymorpha*, the mycelium was too dense to allow imaging of the first steps of appressorial development. To image the “penetration peg” and the “haustorium-like growth” stages in *X. polymorpha*, pictures were taken after on-top-growing mycelium removal. For each appressorium imaged, arrows indicate the contact points and the penetration peg corresponding to the haustorium-like growth (*) in the third column; for *X. polymorpha*, arrows point to every observable penetration peg. Last column (**right**) shows in-depth intra-cellophane mycelial development. For *X. polymorpha*, this step is already represented in the previous “haustorium-like growth” column. Scale bar = 10µm. Magnification in the upper insets is 1.7×.

Small pieces (1 cm^2^) of cellophane covered with spores or mycelium were cut with a scalpel and mounted in water in between standard microscopic slides and coverslips. For every fungus tested, removal of the distal part of the thallus was performed in order to image the intra-cellophane mycelial growth in the absence of the above thallus, which obstructs microscopic observations. This corresponds to the last column in Figure 1 and Figure 2. Due to the significant thickness of *Xylaria polymorpha* mycelium, the penetration pegs, the haustorium-like growth and the intra-cellophane growth were observed only after removal of the mycelium growing on the top of the cellophane layer (Figure 2). Images were taken with an inverted microscope, Leica DMI6000 (Wetzlar, Germany); sCMOS camera; DIC filter; LED EL6000; Imagoseine Imaging Facility: https://imagoseine.ijm.fr/676/accueil.htm. The images were analyzed with Fiji (1.52p version) [16].

## 3. Results

### 3.1. Cellophane for In Vitro Penetration Assays

Cellophane is a transparent film made of cellulose, the main component, along with hemicellulose of plant cell walls. It presents a hard and hydrophilic surface and is well known to induce appressorium development in some filamentous phytopathogenic fungi when they grow on it [17]. We have shown that the saprotrophic fungus *P*. *anserina* was surprisingly capable of differentiating appressoria when grown on cellophane, but we also observed that the presence of glucose in the medium beneath the cellophane layer had an inhibitory effect on appressorium development [8]. We decided to standardize the growth conditions for all the fungi tested and avoided media containing glucose, by choosing the M2 standard medium routinely used in the lab for *P*. *anserina* cultures. This medium includes dextrin as the carbon source and urea as the nitrogen source (see Mat. and Met.). Because of the high diversity of the spores produced by all the fungi tested, and in order to standardize the inoculation conditions, cellophane was inoculated with mycelial implants of each fungus instead of spore suspensions. Indeed, among all the fungi tested, not all the spores produced, whether they be sexual or asexual, were able to germinate on cellophane on top of the M2 medium. For instance, *P*. *anserina* produces asexual spermatia that are unable to germinate, and sexual ascospores that require a specific medium for germination. However, attempts were made to investigate appressorium formation from germinated spores for some of the species (see Section 3.3).

We assessed these conditions for appressorium development on both phytopathogen models *Fusarium graminearum* and *Rhizoctonia solani*, known to penetrate their host in nature and in the lab [7,18]. It is worth noting that *F*. *graminearum* appressorium allows host penetration through stomatal apertures in plants [19]. As observed in Figure 1, both the ascomycete *F*. *graminearum* and the basidiomycete *Rhizoctonia solani* were able to differentiate unicellular appressoria on cellophane on top of M2 medium. In both fungi, we observed similar stages of appressorium development on cellophane as in *P*. *anserina*: (i) growth reorientation of hyphae growing horizontally on cellophane, (ii) contact points made by the hyphae growing towards the cellophane surface and slight swelling of these contact points, (iii) emission of a penetration peg allowing in depth cellophane penetration, (iv) haustorium-like growth, and (v) intra-cellophane mycelial invasion. Significantly, the penetration peg and haustorium-like growth were previously described in *P*. *anserina* as a needle-like hyphae and a palm-like structure, respectively ([8], Figure 2). More details on appressorium differentiation are given below, in Section 3.2.

This first result validated the conditions to test thirty-nine additional *Eumycetes* strains and demonstrated that *F*. *graminearum* appressorium is able to penetrate cellophane. However, these conditions did not allow much growth of saprotrophic *Basidiomycota* (i.e., *Laetiporus sulfureus*, *Phallus impudicus*, *Chlathrus archeri*), minimizing their representation in our study.

### 3.2. Appressorium Development is Widespread in Saprotrophic Dikarya

**Figure 3:**
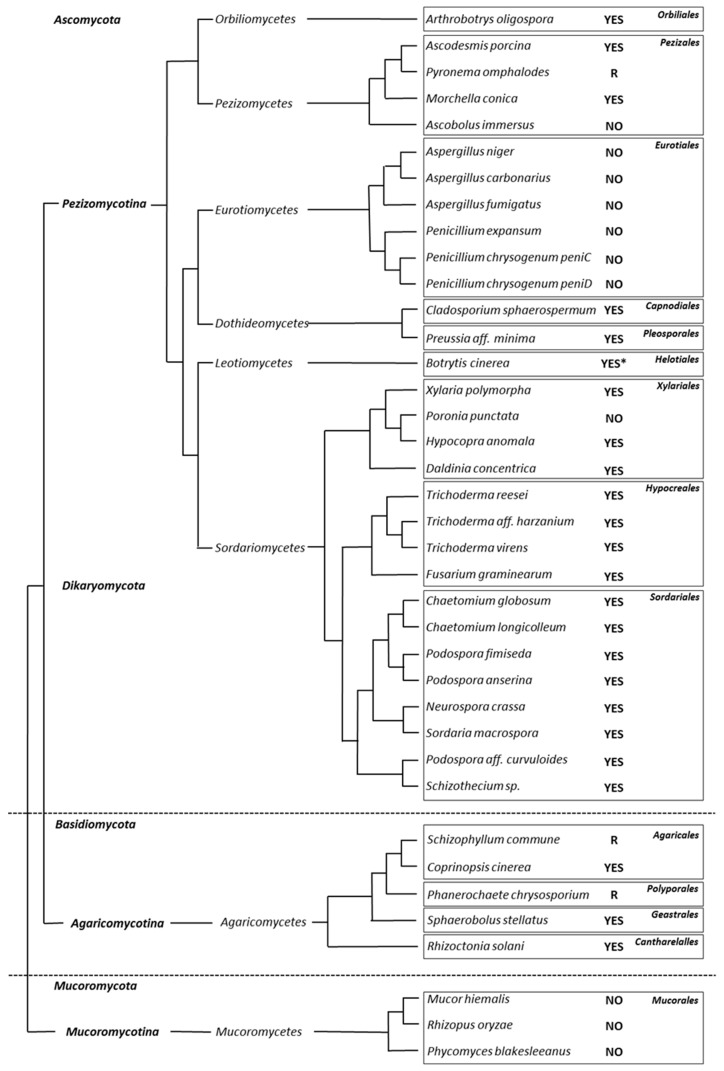
Phylogenetic tree of the 38 *Eumycetes* strains analyzed. “YES”, presence of appressoria; “R”, reorientation of hyphal growth towards cellophane; “NO”, no hyphal growth reorientation and no appressorium developed. This tree was built according to the “MycoCosm Fungal Tree of Life” of the JGI [22].

To address the question of whether saprotrophic *Eumycetes* were in a large part capable of differentiating appressoria when grown on cellophane, we investigated a collection of filamentous fungi available in our lab (Table S1 and Figure 3). Except for the few phytopathogens included in our survey (*B*. *cinerea*, *R*. *solani* and *F*. *graminearum*), all the other examined fungi were either exclusive saprobes, or had a well-defined saprotrophic lifestyle outside their host, like the human pathogen *Aspergillus fumigatus* (*Eurotiomycetes*) [20]. Nevertheless, the *Orbiliomycetes Arthrobotrys oligospora* is both a saprobe and a nematophagous fungus [21]. As reported in Figure 3, most of the tested saprotrophic *Dikarya* were able to differentiate appressoria (“YES” in Figure 3). Figure 2 shows the appressorium differentiation and the intra-cellophane mycelial invasion in a panel of fungi belonging to the main *Dikarya* studied classes: the *Orbiliomycetes*, the *Pezizomycetes*, the *Dothideomycetes*, the *Sordariomycetes* and the *Agaricomycetes*.

**Figure 4:**
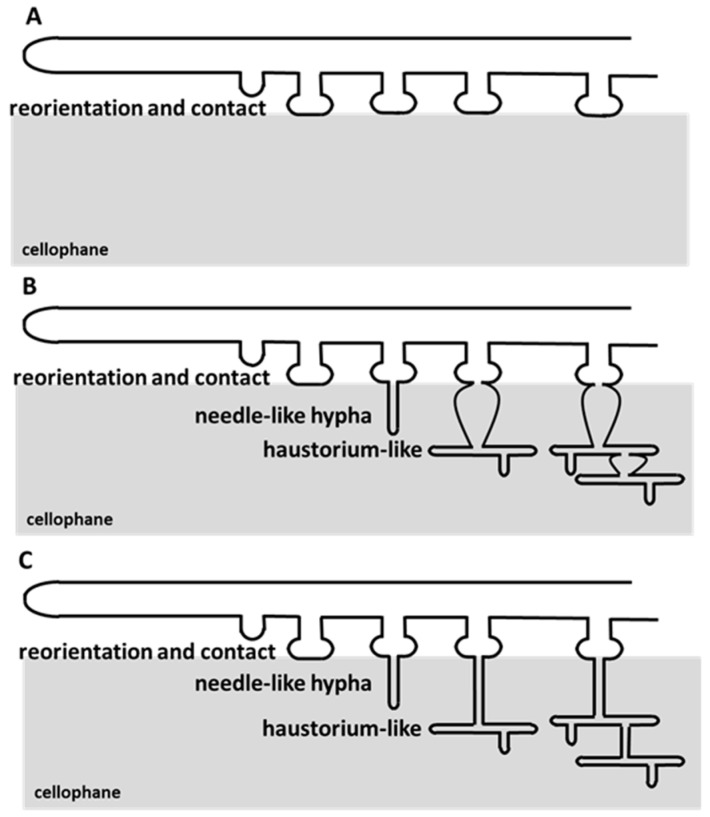
Schematic of appressorial development. (**A**) The fungi, showing clear tropism towards cellophane, reorient hyphal growth towards cellophane and establish contact points at the cellophane surface. (**B**,**C**) The fungi, able to penetrate cellophane, develop appressoria in five steps. (i) They reorient hyphal growth, (ii) establish contact points, (iii) emit a penetration peg in order to breach cellophane, and (iv) starthaustorium-like growth inside the cellophane layer. The haustorium-like enlarges with time and it becomes the base for the emission of more penetration pegs. This process allows invasion (v) and eventual breaching of the cellophane layer. In some species, concurrently to their intra-cellophane development, the penetration peg enlarges and becomes hardly distinguishable from other fungal structures inside the cellophane (**B**).

Since the most central part of thalli was where cellophane penetration and digestion were more advanced and where the penetration process was hardly discernible, we imaged distal parts of thalli to identify appressoria in their early stage of development. Appressorium development followed the pattern previously described above and shown in Figure 1, Figure 2 and Figure 4: (i) hyphal reorientation, (ii) contact point, (iii) penetration peg, (iv) haustorium-like growth, (v) intra-cellophane growth. Appressoria were numerous and easily found in the first centimeter from the edge of thalli. Given the heterogeneous distribution of appressoria on thalli, precise quantification of their number was not possible. However, as shown in Figure 1 and Figure 2, we could often observe several contact points giving rise to a penetration peg and then eventually to haustorium-like growth per 2.5 × 10^3^ µm^2^ image (column 2; 50 × 50 µm^2^). And in Figure 1, for instance, two appressoria for *F*. *graminearum* and four appressoria for *R*. *solani* were observed in the 2.5 × 10^3^-µm^2^ image column 4. Although the morphologies of the haustorium-like growth and of the mycelium invading the cellophane were different between species, appressorium development in each species was really stereotyped: the size of the penetration peg, the shape of the haustorium-like growth as well as the shape of the invading mycelium being quite similar from one appressorium to the other. The size of the penetration peg, estimated as the distance between the contact point and the haustorium-like growth, ranged from 5 µm (*F*. *graminearum*) to 15 µm (*N*. *crassa*), with most species showing a penetration peg approximately 10 µm length. The haustorium-like growth was defined as the initial stage, from which the intra-cellophane growth begins, but both stages are part of a continuous process and the haustorium-like enlarged with time.

In the same way, we observed appressoria in industrial or research-relevant species such as *Trichoderma reesei*, *Neurospora crassa*, *Sordaria macrospora* and *Coprinopsis cinerea*. Indeed, along with *P*. *anserina*, these filamentous fungi are all famous model organisms grown worldwide in laboratories and *T*. *reesei* is widely used in industry.

### 3.3. Mucorales and Eurotiales Do Not Penetrate Cellophane

**Figure 5:**
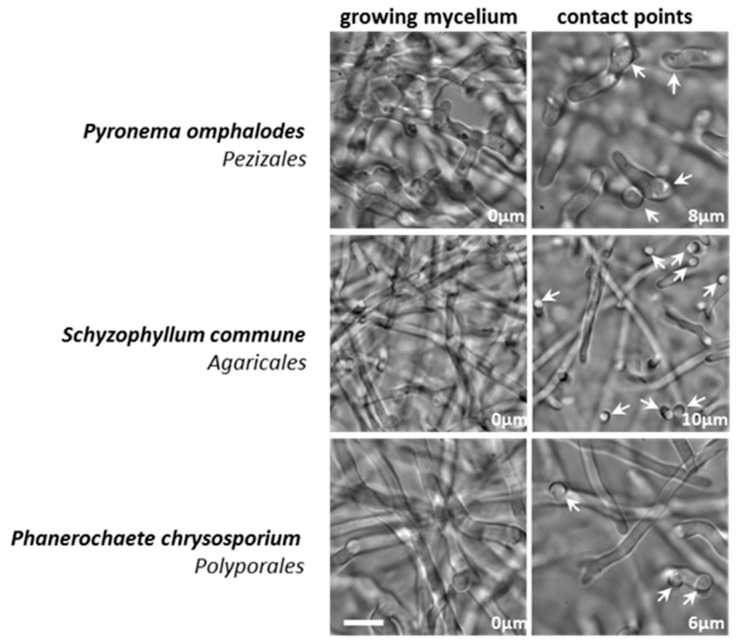
Reorientation and contact points without cellophane penetration. The *Pezizomycetes Pyronema omphalodes*, as well as the *Agaricomycetes Schizophyllum commune* and *Phanerochaete chrysosporium* exert clear tropism towards cellophane. Both columns represent different z-planes of the same field of view. The distances from the initial z-plane are indicated. **Left** column: mycelium growing on top of the cellophane layer. **Right** column: contact points (arrows) observed following hyphal growth reorientation. Scale bar = 10µm.

Some species were not able to differentiate appressoria, but we could observe the clear tropism of hyphal growth towards the cellophane surface. This was the case for the *Pezizomycetes Pyronema omphalodes* and the basidiomycetes white rots *Schizophyllum commune* and *Phanerochaete chrysosporium* (Figure 5). For these three species, we could observe hyphae growing towards the cellophane surface and establishing contacts with the surface, but we did not observe any cellophane penetration nor intra-cellophane growth (see also schematic drawing Figure 4A). On the contrary, several species did not show any tropism towards cellophane nor appressorium differentiation. The three *Mucoromycotina* tested, *Mucor hiemalis*, *Rhizopus oryzae* and *Phycomyces blakesleeanus*, were able to efficiently grow on cellophane but not to penetrate it. Similarly, none of the *Eurotiomycetes* grown on cellophane effectively penetrated it. To investigate this latter clade, we examined two different isolates of *Penicillium chrysogenum*, two further species of the *Penicillium* genus as well as three species of *Aspergilli*. Among them, the human pathogen *A*. *fumigatus* was included—which, when found in nature, is exclusively saprotrophic [20]. We then assayed whether these latter species lacking appressorial development when mycelium was inoculated on top of cellophane, actually develop appressoria directly following spore germination as most pathogenic fungi do (e.g., *B*. *cinerea*, *M*. *oryzae*, *F*. *graminearum*, *B*. *bassiana*) [23]. We therefore investigated appressorial development on germlings in two species able to develop appressoria, *N*. *crassa* and *C*. *cinerea*, and five species lacking appressorial development, *P*. *expansum*, *A*. *carbonarius*, *A*. *niger* (*Eurotiales*), *M*. *hiemalis* and *R*. *oryzae* (*Mucorales*). In all the species tested, the spores did germinate but no appressorium developed from germlings (data not shown).

### 3.4. A. oligospora Differentiates Unicellular Appressoria and Pluricellular Infection Cushions

**Figure 6:**
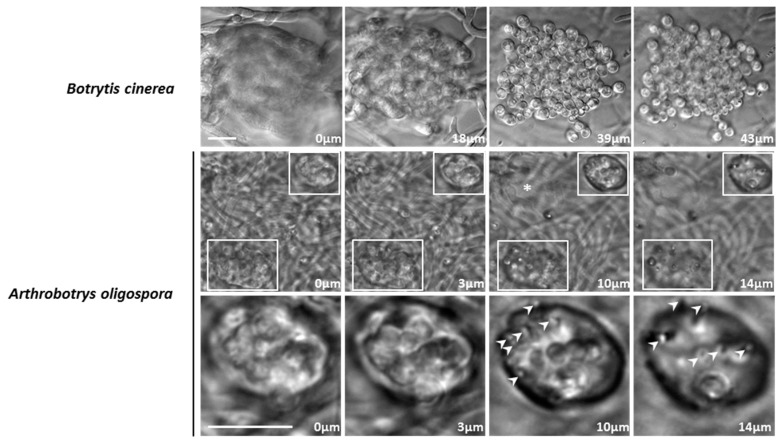
*Botrytis Cinerea* and *Orbiliomycetes Arthrobotrys oligospora* develop infection cushions. From left to right, the four columns represent successive z-planes of the same field of view. The distances from the initial z-plane are indicated. **Upper** row: when grown on cellophane, *B*. *cinerea* develops penetration cushions composed of a bundle of hyphae growing together transversally to the cellophane layer. **Second** row: comparably to *B*. *cinerea*, in *A*. *oligospora*, bundles of hyphae (from 2 to 20) can grow transversally to the cellophane layer, creating an infection cushion (**upper** and **bottom** frames). **The last** row corresponds to the 3× magnification of the infection cushion in the upper frame. Penetration pegs (arrow heads) at the tip of the hyphae compose the infection cushions. (*) Haustorium-like growth. In contrast to *B*. *cinerea*, hyphae of *A*. *oligospora* can be observed inside the cellophane layer. Scale bar = 10 µm.

The unicellular appressorium is not the only penetration structure found in phytopathogenic fungi. Species like *Rhizoctonia sp*., *Sclerotium sp*. (*Basidiomycota*), *Sclerotinia sp*. and *B*. *cinerea* (*Ascomycota*) can differentiate multicellular structures devoted to plant penetration, called infection cushions (see *B*. *cinerea* in Figure 6). One characteristic of the infection cushion is the formation of a bundle of hyphae growing transversally towards the host cuticle or the cell wall allowing penetration of the host [14]. In our assay conditions, we could observe the formation of infection cushions but no unicellular appressorium in *B*. *cinerea*. This was likely due to our inoculation method, using a mycelial implant rather than a conidial suspension (Figure 6). Indeed, in *B*. *cinerea*, the type of infection structure observed depends on the inoculation method [24]. Noteworthy, intra-cellophane invasion by *B*. *cinerea* mycelium was not observed. Strikingly, in addition to the unicellular appressorium differentiated by *A*. *oligospora* (Figure 2), multicellular infection cushions were observed when this fungus was grown on cellophane (Figure 6). The infection cushions in *A*. *oligospora* were composed of bundles of two to approximately twenty hyphae. This number was smaller than the one in *B*. *cinerea* that formed bundles of approximately hundred hyphae (Figure 6). The depth of penetration was greater in *B*. *cinerea* (more than 40 µm, Figure 6) than in *A*. *oligospora* (smaller than 20 µm, Figure 6). This was due to the fact that in the case of *B*. *cinera*, hyphae composing the infection cushion did not invade cellophane, while those of *A*. *oligospora* infection cushions eventually invaded cellophane a few micrometers away from the penetration point. Contrary to *B*. *cinerea* infection cushions, thin and short tips ahead of the hyphae composing the infection cushion were observed in *A*. *oligospora* (arrow heads Figure 6). This showed that the hyphae of the infection cushion in *A*. *oligospora* differentiated a penetration peg, likely to facilitate cellophane penetration.

## 4. Discussion

Host penetration by fungi has long been an important field of research. Due to its central role in the penetration process, the appressorium, the dedicated structure in fungal pathogens as well as in mutualistic fungi, has been thoroughly studied and reviewed [5,7,24,25,26,27]. Recently, we have discovered that the coprophilous fungus *P*. *anserina* is able to differentiate penetration structures that we previously named appressorium-likes [8]. Here, we demonstrate that the capacity to penetrate a solid surface is widely observed in saprotrophic filamentous fungi. Despite the diversity in the morphology of penetration structures, we can define penetration stages shared by all the filamentous fungi that differentiate penetration structures: (i) hyphae or germ tube (when a contaminating spore germinates) growth reorientation, (ii) setting up of a contact point with the surface (regardless of how this contact adheres to the surface and whether this contact swells, is reinforced by melanin, chitin, or if it involves pressure), (iii) differentiation of a penetration peg, (iv) differentiation of a structure similar to the haustorium and enabling nutrient uptake, and (v) development inside the host/the biomass once penetration has occurred. With respect to the broad definition of the appressorium given by Emmett and Parbery [7], we have named these penetration structures in saprotrophic fungi “appressoria”.

As other saprotrophic fungi, *P*. *anserina* is a potent plant biomass degrader and the sequencing of its genome highlighted the impressive set of dedicated enzymes it possesses [3]. Contrary to many *Ascomycota*, *P*. *anserina* harbors an impressive set of genes required for lignin degradation, and it efficiently utilizes lignocellulose as a carbon source [4]. *P*. *anserina* is thus endowed with a complete arsenal allowing efficient plant biomass penetration and degradation. We have shown that the complete lack of cellophane penetration in *PaNox2* mutants correlates with diminished cellulose degradation efficiency in *P*. *anserina*, supporting the idea that both cellulose (cellophane) penetration and cellulose lysis are intimately linked [8]. Hence, efficient biomass deconstruction by saprotrophic fungi may not only rely on enzymatic capacities, but it may also require morphogenetic programs, such as the development of an appressorium. This latter assumption should be further considered while studying plant biomass degradation by fungi.

We have previously shown that all the isolates examined in the *P*. *anserina* species complex differentiate appressoria [27]. In the present paper, we show that in fact many saprotrophic *Dikarya* differentiate appressoria: 64% of the investigated species growing on cellophane (21 out of 33 saprotrophic species). These include species present in every main *Pezizomycotina* clade comprising saprotrophic species and in both *Agaricomycetes* (*Basidiomyota*) orders that we tested, *Agaricales* and *Geastrales*. Moreover, we do not exclude that the species not able to grow or not able to develop appressoria in our specific assay conditions, may actually develop appressoria in other lab conditions or in their natural habitat. For some of these fungi failing to produce penetration structures (*Eurotiales* and *Mucorales*), we further investigated their capacity to develop appressoria from germlings, but the results were similar to the one observed with a mycelial inoculum: none of them developed an appressorium. It is worth noting that in both fungi *C*. *cinerea* and *N*. *crassa*, appressorium development did not occur right after germination of their respective asexual spores, the oïdia and the macroconidia. Hence, to answer the question of whether saprotrophic fungi develop appressoria from “contaminating” spores (sexual or asexual) in the same manner as plant pathogens, will require a thorough investigation of each specific species in germination condition on cellophane or any other surface.

*P*. *anserina* potently grows and develops appressoria on cellophane which presents a hydrophilic surface. In contrast to many plant pathogens, this fungus hardly grows and does not develop appressoria on hydrophobic surfaces [8]. Hence, cellophane was chosen as the standard surface for our penetration tests, and except for three basidiomycetes strains, most of the fungal saprotrophs assayed in this paper grew well on cellophane and eventually penetrated it. Our paper represents the pioneer survey for appressorium development in saprotrophic *Eumycetes*. This work is a stepping stone, and from now on, experimental conditions specifically designed for every single saprotrophic fungus of interest will be required to fully investigate their capacity to develop penetration structures and to understand how this developmental program is regulated. In particular, the nature of the surface to penetrate (hydrophilic vs. hydrophobic), the nutritive medium as well as the inoculation methods will be paramount.

Further studies examining a broader number of saprotrophic *Basidiomycota* may answer the question whether appressorium development is as widespread in this clade as it is in *Ascomycota*. Several *Basidiomycota* plant pathogens are known to develop appressoria. This is the case of the maize pathogen *Ustilago maydis*, and several rusts in the *Pucciniomycetes* class as well as in the *Agaricomycetes R*. *solani* [25,28]. Nowadays, appressoria in these different clades are not considered as homologous structures, but this paradigm is now challenged by our discovery of appressoria all along the phylogenetic tree of *Dikarya*. We previously demonstrated that the conserved set of genes controlling appressorium development in plant pathogens belonging to *Sordariomycetes* (*M*. *oryzae*) and *Leotiomycetes* (*B*. *cinerea*), also controls appressorium development in the saprobe *P*. *anserina* (*Sordariomycetes*). This includes the genes encoding the Fus3/PaMpk2/Pmk1 MAPK pathway components, both Nox complexes Nox1 and Nox2, as well as their regulatory subunits NoxD, NoxR and the tetraspanin Pls1 [9,15,29]. Hence, appressoria in saprotrophic fungi and in pathogenic fungi may be homologous structures. In particular, we want to highlight the fact that Nox2, with its role in the polymerization of the appressorium basal septin ring as well as the remodeling of the actin cytoskeleton (in *M*. *oryzae*) together with its partner Pls1, which is required for cell polarity re-establishment at onset of appressorium development (in *M*. *oryzae*) [10,15,30,31], may both be pivotal components of appressorium development in filamentous fungi. Intriguingly, despite the ability of *Aspergillus fumigatus* to efficiently invade tissues and organs in human patients [20], no specialized penetration structure in this opportunistic pathogen that lacks both Pls1 and Nox2 has been described yet.

Although *Saccharomycotina* and *Taphrinomycotina* include several plant pathogens, such as *Taphrina sp*. (*Taphrinomycotina*), *Eremothecium sp*. (*Saccharomycotina*) as well as human pathogens, such as *Pneumocystis jirovecci* (*Taphrinomycotina*), *Candida albicans* and *Candida glabrata* (*Saccharomycotina*), appressoria have never been described in any of these phyla. However, mycoparasitic yeasts such as *Arthroascus javanensis* or *Saccharomycopsis javanensis* (*Saccharomycotina*) are able to penetrate yeast cells and establish an haustorium inside the host cell [32]. Whether the appressorium of *Pezizomycotina* and this penetration structure in *Saccharomycotina* are homologous structures remains an open question.

In contrast to *Dikarya*, none of the saprotrophic *Mucoromycota* tested in this study differentiates appressoria. Nevertheless, symbiotic *Mucoromycota*, such as *Rhizophagus sp*. as well as entomopathogenic *Entomophtorales* develop appressoria [33,34]. Whether appressoria in *Mucoromycota* are homologous to those of *Dikarya* remains to be addressed.

## 5. Conclusions

Our paper showing that appressorium development is widespread in *Dikarya* opens a new area in the functional study of appressorium development. While in the most studied plant pathogens, the forward genetics approach, the most powerful strategy to uncover new genes, is rarely an option, genetic dissection of appressorium development in filamentous fungi can now be undertaken in saprotrophic model species such as *N*. *crassa*, *S*. *macrospora* and *P*. *anserina*, all of them being powerful genetics model organisms. On a broader scale, the discovery of appressorium development in a wide array of fungi reshuffles the cards of the origin of the appressorium during the evolution of *Eumycetes*. Hence, functional studies of appressorium development in a wide array of *Dikarya* as well as in *Mucoromycota* will be required to answer the question of the homology of penetration structures in *Eumycetes*.

## Supplementary Materials

The following are available online at https://www.mdpi.com/2309-608X/5/3/72/s1, Table S1. Strains list.
