# Supplementary material for: Appressorium: The Breakthrough in Dikarya
Source: J Fungi (Basel). 2019 Aug 3;5(3):72. doi: 10.3390/jof5030072 (PMC6787622; doi:10.3390/jof5030072)
Supplement: Supplementary file 1 [file jof-05-00072-s001.pdf]

class	order	species	Strain ref.	trophic mode	particularity
Orbiliomycetes	*Orbiliales*	*Arthrobotrys oligospora*	CBS 115.81	saprotroph	nematophagous
Pezizomycetes	*Pezizales*	*Ascodesmis porcina*	PSN5	saprotroph	coprophilous
		Pyronema omphaloïdes	CBS 283.31	saprotroph	soil-borne
		Morchella conica	PSN22	saprotroph	soil-borne
		Ascobolus immersus	FB17 (mat-)	saprotroph	coprophilous
Eurotiomycetes	*Eurotiales*	*Aspergillus niger*	AN26	saprotroph	ubiquitous
		Aspergillus carbonarius	AC19	saprotroph	ubiquitous
		Aspergillus fumigatus	PSN184	saprotroph	human pathogen
		Penicillium expansum	PSN81	saprotroph	ubiquitous
		Penicillium chrysogenum	peni C	saprotroph	ubiquitous
		Penicillium chrysogenum	peni D	saprotroph	ubiquitous
Dothideomycetes	*Capnodiales*	*Cladosporium sphaerospermum*	BCCM-IHEM 3169	saprotroph	habitats
	Pleosporales	*Preussia aff. minima*	PSN296	saprotroph	coprophilous
Leotiomycetes	*Helotiales*	*Botrytis cinerea*	B05.10	phytopathogen	necrotrophic
Sordariomycetes	*Xylariales*	*Xylaria polymorpha*	PSN28	saprotroph	wood degrader
		Poronia punctata	CBS 656,78	saprotroph	wood degrader
		Hypocopra anomala	CBS 124649	saprotroph	wood degrader
		Daldinia concentrica	PSN218	saprotroph	wood degrader
	Hypocreales	*Trichoderma reesei*	CBS 383.78	saprotroph	ubiquitous
		Trichoderma aff. harzanium	PSN262	saprotroph	mycoparasite
		Trichoderma virens	CBS 430.54	saprotroph	mycoparasite
		Fusarium graminearum	PH1	phytopathogen	hemibiotrophic
	Sordariales	*Chaetomium globosum*	DSMZ 62,110	saprotroph	coprophilous
		Chaetomium longicolleum	CBS 119.57	saprotroph	coprophilous
		Podospora fimiseda	CBS 990.96	saprotroph	coprophilous
		Podospora anserina	“Big S” strain	saprotroph	coprophilous
		Neurospora crassa	OR74A	saprotroph	ubiquitous
		Sordaria macrospora	000	saprotroph	coprophilous
		Podospora aff. curvuloides	PSN258	saprotroph	coprophilous
		Schizothecium sp.	PSN244	saprotroph	coprophilous
Agaricomycetes	*Agaricales*	*Schizophyllum commune*	PSN11	saprotroph	wood degrader
		Coprinopsis cinerea	H5	saprotroph	soil-borne
	Polyporales	*Phanerochaete chrysosporium*	RP78	saprotroph	wood degrader
	Geastrales	*Sphaerobolus stellatus*	DSM1028	saprotroph	wood degrader
	Cantharelalles	*Rhyzoctonia solani*	SB1	phytopathogen	necrotrophic
Mucoromycetes	*Mucorales*	*Mucor hiemalis*	PSN56	saprotroph	ubiquitous
		Rhizopus oryzae	RA 99880	saprotroph	ubiquitous
		Phycomyces blakesleeanus	NRRL1555 (mat-)	saprotroph	ubiquitous

Table S1 : strains list
